# MiR‐199b‐5p inhibits osteogenic differentiation in ligamentum flavum cells by targeting JAG1 and modulating the Notch signalling pathway

**DOI:** 10.1111/jcmm.13047

**Published:** 2016-12-13

**Authors:** Xiaochen Qu, Zhongqiang Chen, Dongwei Fan, Chuiguo Sun, Yan Zeng, Zhaoqing Guo, Qiang Qi, Weishi Li

**Affiliations:** ^1^ Department of OrthopaedicsPeking University Third HospitalBeijingChina

**Keywords:** ossification of the ligamentum flavum, miR‐199b‐5p, osteogenic differentiation, JAG1, Notch signalling pathway

## Abstract

Ossification of the ligamentum flavum (OLF) is a pathology almost only reported in East Asian countries. The leading cause of OLF is thoracic spinal canal stenosis and myelopathy. In this study, the role of miR‐199b‐5p and jagged 1 (JAG1) in primary ligamentum flavum cell osteogenesis was examined. MiR‐199b‐5p was found to be down‐regulated during osteogenic differentiation in ligamentum flavum cells, while miR‐199b‐5p overexpression inhibited osteogenic differentiation. In addition, JAG1 was found to be up‐regulated during osteogenic differentiation in ligamentum flavum cells, while JAG1 knockdown *via* RNA interference caused an inhibition of Notch signalling and osteogenic differentiation. Moreover, target prediction analysis and dual luciferase reporter assays supported the notion that JAG1 was a direct target of miR‐199b‐5p, with miR‐199b‐5p found to down‐regulate both JAG1 and Notch. Further, JAG1 knockdown was demonstrated to block the effect of miR‐199b‐5p inhibition. These findings imply that miR‐199b‐5p performs an inhibitory role in osteogenic differentiation in ligamentum flavum cells by potentially targeting JAG1 and influencing the Notch signalling pathway.

## Introduction

OLF is a pathology almost exclusively reported in Eastern Asian countries. OLF primarily occurs in the thoracolumbar spine and is the main cause of thoracic spinal canal stenosis and myelopathy 1, 2. In the past several years, many studies have examined OLF development and progression at both histopathological and cellular levels. While these studies identified potential contributing factors, such as mechanical 3, 4, 5, 6, metabolic 7, 8, degenerative 9 and genetic factors 10, 11, OLF development and progression continues to be inadequately understood.

Today, OLF and ossification of the posterior longitudinal ligament (OPLL) are collectively referred to as ossification of spinal ligament (OSL) disorders, where both are characterized by endochondral ossification 12. While various signalling pathways have been connected with the regulation of osteogenic differentiation in general, some have also been associated with OSL pathogenesis 6, 7, 13, 14, 15, 16. Of these, the Notch signalling pathway, which influences proliferation, differentiation and mineralization in osteoblasts, has been found to encourage osteogenic differentiation in ligamentum flavum cells 17, 18.

One potential way that osteogenic differentiation is modulated is through MicroRNAs (miRNAs). MiRNAs, which comprise a substantial family of small (18–24 nucleotides), single‐stranded non‐coding RNAs, function in the regulation of mammalian cell gene expression. MiRNAs regulate a target mRNA by binding its 3′‐untranslated region (UTR) and subsequently mediating its degradation *via* the RNA‐induced silencing complex (RISC) 19. MiRNAs regulate a variety of physiological and pathological processes, with previous studies showing that particular miRNAs have the potential to positively or negatively regulate osteogenesis or osteoclastogenesis 20. Additionally, recent study implicated several miRNAs as serving pivotal roles in OPLL onset and progression 21. Of these identified miRNAs, miR‐199b‐5p, which is one of the top 10 down‐regulated miRNAs, was predicted to regulate JAG1, a significant Notch signalling pathway ligand. However, another recent study found that miR‐199b‐5p was up‐regulated in the osteogenic differentiation in the bone marrow stromal cells (BMSCs) 22. So far as we know, whether miR‐199b‐5p is involved in the process of OLF has not been investigated.

In this study, the role of miR‐199b‐5p and JAG1 was further characterized during osteogenic differentiation in ligamentum flavum cells. Our results implied that miR‐199b‐5p, which is down‐regulated during osteogenic differentiation in ligamentum flavum cells, inhibits the differentiation process by targeting JAG1 and affecting the Notch signalling pathway.

## Materials and methods

### Patient specimens

**Figure 1:**
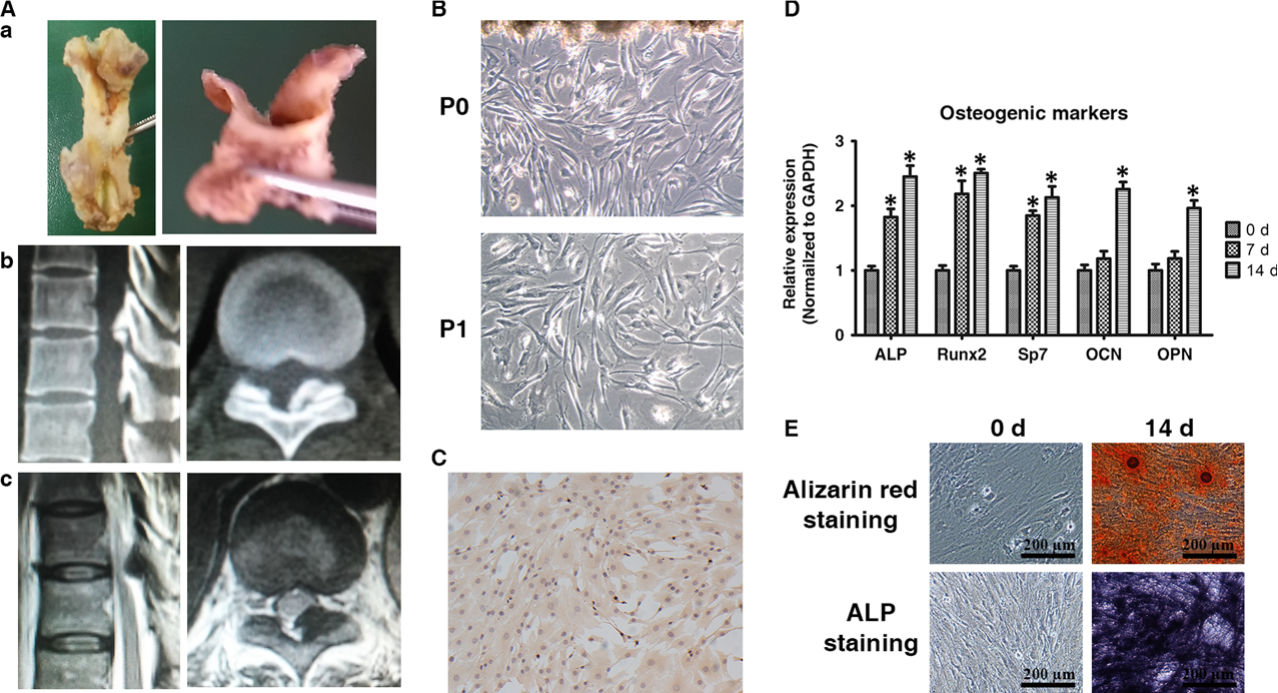
Identification and characterization of OLF patient ligamentum flavum cells. (**A**) Representative OLF patient ligamentum flavum sample obtained *via en bloc* resection of the lamina and ligamentum flavum. General sample (a), computed tomography (b) and magnetic resonance imaging (c), including sagittal and cross‐plane scans of the ossification area at the corresponding position. (**B**) Representative morphology of P0 and P1 OLF patient ligamentum flavum cells. (**C**) Immunocytochemical detection of vimentin in OLF cells; scale bar represents 200 μm. (**D**) qRT‐PCR analysis of five osteogenic markers in OLF patient ligamentum flavum cells; *P < 0.05 C compared with day 0. (**E**) ALP activity and Alizarin red staining of OLF patient ligamentum flavum cells; scale bar represents 200 μm.

Patient specimens were obtained from the biobank of the Department of Orthopedics, Peking University Third Hospital with approval of the Ethics Committee for Human Subjects at Peking University Third Hospital. OLF patients who visited the orthopaedic clinic and provided written informed consent for the study were utilized. Specialists diagnosed OLF based on clinical symptoms and radiological examination as previously described 23 (Table 1). Ligamentum flavum samples were obtained from OLF patients during spinal surgery *via en bloc* resection of the lamina and ligamentum flavum (Fig. 1A) as previously described 24.

**Table 1:** Clinical information of OLF patients

Number	Sex	Age (years)	Diagnosis	Level
OLF‐1	M	49	OLF	T11/12
OLF‐2	F	52	OLF	T9/11
OLF‐3	M	46	OLF	T8/11
OLF‐4	F	55	OLF	T11/L1

### Cell cultures and osteogenic differentiation

Ligaments (approximately 0.5–1 cm^2^) were aseptically harvested from patients during surgery and rinsed with phosphate‐buffered saline (PBS), while surrounding tissues were removed under a dissecting microscope to avoid possible osteogenic cell contamination. The collected ligaments were minced into approximately 0.5‐mm^3^ pieces and digested using 0.25% trypsin, followed by 250 U/ml type I collagenase (Sigma‐Aldrich, St. Louis, MO, USA). The specimen were washed with serum‐containing medium and placed in 100‐mm culturing dishes containing Dulbecco's Modified Eagle's medium (DMEM; Gibco, Grand Island, NY, USA) supplemented with 10% foetal bovine serum (Gibco), 100 U/ml penicillin G sodium and 100 mg/ml streptomycin sulphate in a humidified atmosphere with 5% CO_2_ at 37°C. Explant‐derived cells derived were harvested using 0.25% trypsin for further passaging, with passages (P) 2 and 3 used for subsequent experimentation. To induce osteogenic differentiation, cells were cultured in osteogenic medium consisting of DMEM supplemented with 50 μM ascorbic acid (Sigma‐Aldrich), 10 mM β‐glycerophosphate (Sigma‐Aldrich) and 10^−8^ M dexamethasone (Sigma‐Aldrich).

### Immunocytochemical staining of vimentin

To evaluate vimentin expression, P2 cells were grown on 2 × 2 cm^2^ slides in six‐well plates at a density of 1 × 10^5^ cells/well. At confluency, cells were fixed at room temperature with 4% paraformaldehyde, washed three times with PBS and permeabilized with 2% Triton X‐100 in PBS. Non‐specific antibody binding was blocked with 3% normal goat serum in PBS, followed by 2‐hrs incubation at 37°C with primary anti‐vimentin antibody (1:250, ab92547; Abcam, Cambridge, MA, USA). Samples were subsequently incubated for 30 min. at room temperature with biotinylated goat anti‐rabbit IgG (31460; Thermo, Waltham, MA, USA), followed by visualization with DAB solution. Excess DAB was removed with a water rinse, and the samples were counterstained with hematoxylin to visualize the nuclei. Negative control samples were incubated with PBS instead of primary antibody under the same conditions.

### Quantitative real‐time polymerase chain reaction (qRT‐PCR) analysis

Total RNA was isolated using Trizol (Invitrogen, Carlsbad, CA, USA). Reverse transcription and qRT‐PCR for miR‐199b‐5p were performed using a miDETECTA Track™ miRNA qRT‐PCR Starter kit (RiboBio, Guangzhou, China) according to the manufacturer's instructions on a BioRad IQ5 system. Each value was normalized to that of RnU6. To examine JAG1, GSK3B, CTNNB1 and five osteogenic markers gene expression levels, reverse transcription and qRT‐PCR were carried out as described previously 18. Expression levels were normalized to glyceraldehyde 3‐phosphate dehydrogenase (GAPDH), and relative gene expression levels were calculated using the 2^−ΔΔCt^ method. All experiments were performed in triplicate. The primers for miR‐199b‐5p and RnU6 were purchased from RiboBio, with the other primers described in Table 2.

**Table 2:** Primer sequences for qRT‐PCR

Gene	Forward primer (5′–3′)	Reverse primer (5′–3′)
JAG1	TCACGGGAAGTGCAAGAGTC	GTTTCACAGTAGGCCCCCTC
ALP	CCAAGGACGCTGGGAAATCT	TATGCATGAGCTGGTAGGCG
Runx2	GCGCATTCCTCATCCCAGTA	GGCTCAGGTAGGAGGGGTAA
Osterix	AAACCCAAGGCAGTGGGAAA	TGCCCCCATATCCACCACTA
OCN	ATGAGAGCCCTCACACTCCT	CTTGGACACAAAGGCTGCAC
OPN	CATACAAGGCCATCCCCGTT	GGGTTTCAGCACTCTGGTCA
GSK3B	CGAGACACACCTGCACTCTT	TTAGCATCTGACGCTGCTGT
CTNNB1	ATGACTCGAGCTCAGAGGGT	ATTGCACGTGTGGCAAGTTC
GAPDH	TCAAGGCTGAGAACGGGAAG	TGGACTCCACGACGTACTCA

### Western blot analysis

Cell lysates were obtained using RIPA lysis buffer (Beyotime, Shanghai, China) containing 10 mM phenylmethylsulphonylfluoride as a protease inhibitor (PMSF; Beyotime), and 50 μg of total protein was separated in a Bis‐Tris polyacrylamide gel and transferred onto a nitrocellulose membrane. The membrane was then incubated in 5% bovine serum albumin (BSA) containing primary rabbit‐anti‐human polyclonal antibodies at 4°C overnight. Next, samples were incubated with IRDye^®^ 800CW goat‐anti‐rabbit antibody at room temperature for 1 hr and visualized *via* chemiluminescence with an infrared laser scanning system (Odyssey Licor, Lincoln, NE, USA). The following primary rabbit‐anti‐human antibodies were used: anti‐JAG1 (1:1000, ab109536; Abcam); anti‐Runx2 (1:1000, ab23981; Abcam); anti‐Sp7/Osterix (1:2000, ab22552; Abcam); anti‐ALP (1:2000, ab95462; Abcam); anti‐OCN (1:500, ab93876; Abcam); anti‐OPN (1:1000, ab8448; Abcam); anti‐cleaved‐Notch 1 (V1754) (1:500, YC0067; Immunoway, Newark, DE, USA); anti‐cleaved‐Notch 2 (D1733) (1:500, YC0069; Immunoway); anti‐β‐Catenin (1:5000, ab32572; Abcam); anti‐ GSK‐3β (1:5000, ab32391; Abcam); and anti‐GAPDH (1:2500, ab9485; Abcam).

### Alkaline phosphatase (ALP) activity assay and Alizarin red staining

To quantify osteogenic differentiation in ligamentum flavum cells, an ALP assay, which is used as an early marker of osteogenic differentiation, and Alizarin red staining, which detects mineralization during the later stages of bone formation, were performed. Cells were seeded in six‐well plate at a density of 1 × 10^5^ cells/well and cultured in osteogenic medium for 0 or 14 days. ALP activity was determined using an ALP activity staining kit (GMS80033.1; GENMED Scientifics, Shanghai, China), and mineralization was assessed using an Alizarin Red S kit (GMS80046.3; GENMED Scientifics).

### MiRNA/siRNA transfection

Ligamentum flavum cells were transfected with miR‐199b‐5p mimics or inhibitor (20 nM), with non‐specific microRNA (miR‐NC; RiboBio) or inhibitor (miR‐NC‐I; RiboBio) used as a negative control; or siRNA targeting JAG1, GSK3B or CTNNB1 (50 nM), with non‐targeting siRNA (siNC; RiboBio) used as negative control, using Lipofectamine^®^ 2000 Transfection Reagent (Life Technologies, New York, NY, USA) according to the manufacturer's instructions.

### Luciferase constructs and reporter assay

The DNA sequences of JAG1, transforming growth factor beta 2 (TGFB2) and SRY‐box 6 (SOX6) 3′‐UTR, were amplified by PCR using HEK293T genomic DNA as a template. The amplified DNA sequences were inserted into pmiR‐RB‐REPORT™ vectors (RiboBio) to generate wild‐type (WT) JAG1, TGFB2 and SOX6 3′‐UTR, with a mutated (MUT) JAG1 3′‐UTR luciferase vector generated using site‐directed mutagenesis. For the reporter assay, HEK293T cells were cultured in a 96‐well plate with 1.5 × 10^4^ cells/well in 100 μl of culture medium/well for 24 hrs. Cells were then co‐transfected with 50 nM miR‐199b‐5p mimic and 100 ng of vector per well and cultured in fresh medium for an additional 48 hrs. The luciferase reporter assay was carried out using the Dual‐Glo^®^ Luciferase Assay System (Promega, Madison, WI, USA) according to the manufacturer's instructions, and luminescence was quantified using a Veritas™ 9100‐002 luminometer (Promega).

### Statistical analysis

Data are presented as a mean ± S.D. Comparisons between groups were analysed *via* two‐tailed *t*‐test using SPSS 17.0 (Chicago, IL, USA), and statistical significance was defined as *P* < 0.05.

## Results

### Identification and characterization of OLF patient ligamentum flavum cells

OLF patient ligamentum flavum‐derived P0 and P1 cells showed fibroblast‐like morphological characteristics to include a big, flat cell body with multiple spindle‐shaped or star‐shaped processes (Fig. 1B). In addition to these morphological attributes, vimentin, a kind of intermediate filament that is highly expressed in fibroblasts and indicative of a fibroblastic phenotype, was also examined immunocytochemically. These results displayed positive cytoplasmic staining for vimentin, which was consistent with the observed morphological features (Fig. 1C).

Following fibroblast establishment, osteogenic differentiation was induced and assessed *via* osteogenic markers and ALP activity and mineralization assays (Fig. 1D and E), with significant increases in all of the determinants noted. Runx2 and Osterix expression levels were increased by day 7 and maintained until day 14. ALP, an early osteogenic marker, and OCN and OPN, late osteogenic markers, were also notably up‐regulated during ossification in a time‐ordered manner. Additionally, ALP activity and the formation of mineralized nodules were also observed by day 14.

### MiR‐199b‐5p inhibits osteogenesis in ligamentum flavum cells

**Figure 2:**
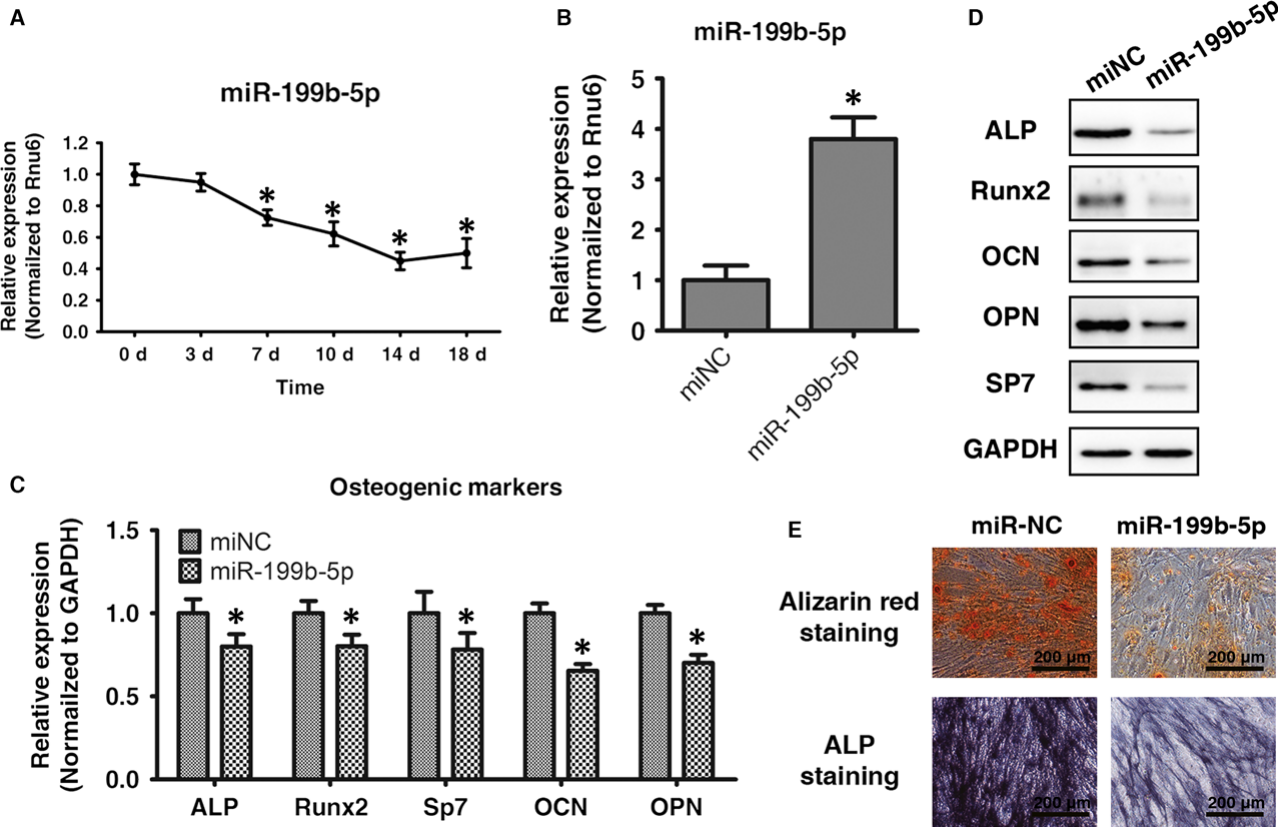
MiR‐199b‐5p inhibits osteogenesis in ligamentum flavum cells. (**A**) Endogenous miR‐199b‐5p expression levels were measured *via* qRT‐PCR at different time points during osteogenic differentiation in ligamentum flavum cells; *P < 0.05 compared with day 0. (**B**) MiR‐199b‐5p expression assessed *via* qRT‐PCR in ligamentum flavum cells transfected with miRNA mimics; *P < 0.05 compared with miR‐NC group. (**C**) qRT‐PCR analysis of osteogenic marker genes after miR‐199b‐5p overexpression at day 14; *P < 0.05 compared with miR‐NC group. (**D**) Western blot analysis of osteogenic marker protein expression after miR‐199b‐5p overexpression at day 14. (**E**) ALP staining and Alizarin Red staining at day 14 showed inhibited ALP activity and calcification after miR‐199b‐5p overexpression when compared with miR‐NC; scale bar represents 200 μm.

When examining miR‐199b‐5p expression during osteogenesis, expression levels were seen to decrease by day 3 relative to day 0 and continued to decline until day 14. These results suggested that miR‐199b‐5p may negatively regulate osteogenic differentiation in ligamentum flavum cells (Fig. 2A). To further clarify the role of miR‐199b‐5p in the regulation of osteogenic differentiation in ligamentum flavum cells, cells were transfected with synthetic miR‐199b‐5p mimics, and osteogenic capacity was examined. Following transfection, intracellular miR‐199b‐5p levels were markedly up‐regulated (Fig. 2B) and osteogenic differentiation was significantly inhibited, as indicated by a reduced expression of osteogenic transcription factors (Runx2 and SP7) and osteoblastic markers (ALP, OPN and OCN) and reduced ALP and Alizarin red staining (Fig. 2C–E).

### JAG1 knockdown down‐regulates Notch signalling and inhibits osteogenic differentiation in ligamentum flavum cells

**Figure 3:**
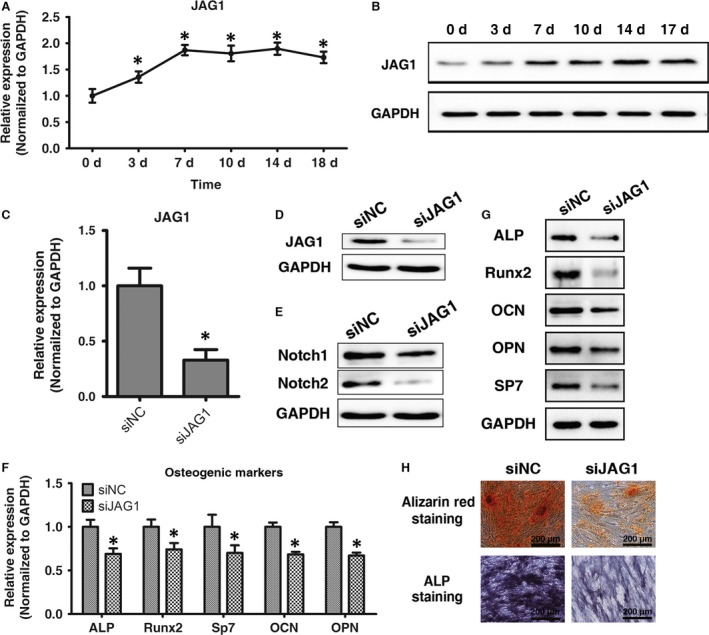
JAG1 knockdown down‐regulates Notch signalling and inhibits osteogenic differentiation in ligamentum flavum cells. (**A, B**) JAG1 mRNA and protein expression levels examined *via* qRT‐PCR and Western blot at different time points during osteogenic differentiation in ligamentum flavum cells; *P < 0.05 compared with day 0. (**C, D**) JAG1 mRNA and protein expression level examined *via* qRT‐PCR and Western blot following siJAG1 transfection in ligamentum flavum cells; *P < 0.05 Compared with si‐NC group. (**E**) Notch1 and Notch2 protein expression levels examined *via* Western blot in siJAG1 transfected ligamentum flavum cells. (**F, G**) Osteogenic marker mRNA and protein expression examined *via* qRT‐PCR and Western blot at day 14 after JAG1 knockdown; *P < 0.05 Compared with si‐NC group. (**H**) ALP staining and Alizarin Red staining at day 14 showed inhibited ALP activity and calcification following JAG1 knockdown when compared with miR‐NC group; scale bar represents 200 μm.

JAG1 mRNA and protein expression levels were determined by qRT‐PCR and Western blot analyses. JAG1 mRNA expression levels increased at day 7 and remained elevated to day 17 (Fig. 3A), while protein levels were continually increased to day 17 (Fig. 3B). These results showed an inverse trend relative to miR‐199b‐5p levels, thus suggesting that JAG1 is post‐transcriptionally regulated during osteogenic differentiation in ligamentum flavum cells. To examine the functional effects of JAG1 on osteogenic differentiation in ligamentum flavum cells, siRNA‐induced JAG1 knockdown was employed and significantly reduced both JAG1 mRNA and protein expression (Fig. 3C and D). JAG1 is a vital Notch ligand and plays a vital role in the Notch signalling pathway, and thus Notch expression was also examined. Following JAG1 knockdown, Notch2 expression was greatly diminished, but not Notch1 (Fig. 3E). Furthermore, JAG1 knockdown inhibited osteogenic differentiation in ligamentum flavum cells, as indicated by reduced RUNX2, SP7, ALP, OPN and OCN expression (Fig. 3F and G) and reduced ALP and Alizarin red staining (Fig. 3H).

### MiR‐199b‐5p directly targets JAG1 and affects Notch signalling

**Figure 4:**
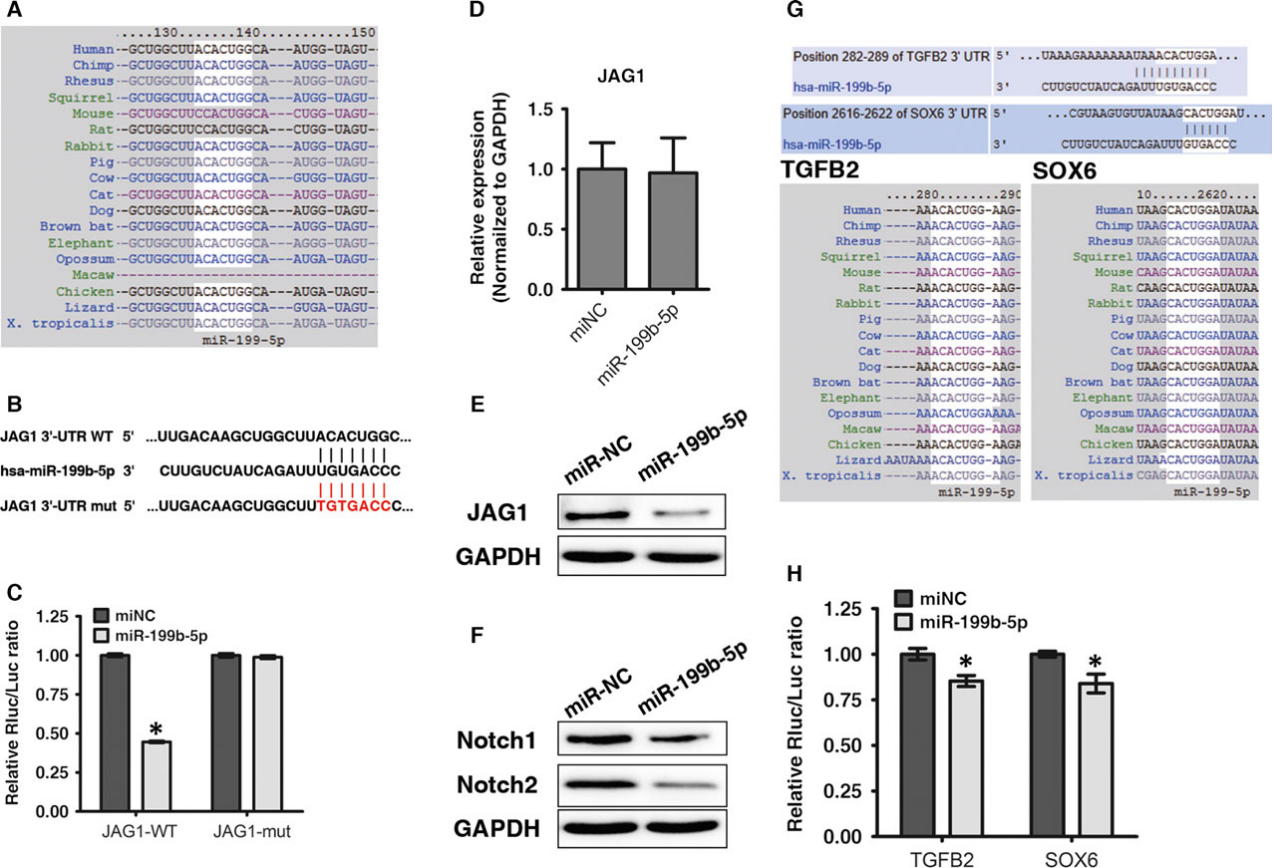
MiR‐199b‐5p directly targets JAG1 and affects Notch signalling. (**A**) The miR‐199b‐5p binding site in the JAG1 3′‐UTR is highly conserved among vertebrates. (**B**) A schematic diagram indicating the wild‐type and mutated‐type miR‐199b‐5p binding sites in the JAG1 3′‐UTR. (**C**) A wild‐type (WT) JAG1 3′‐UTR or a mutant (MUT) JAG1 3′‐UTR reporter plasmid was co‐transfected into HEK293T cells with either miR‐199b‐5p or mi‐NC and fluorescence was quantified; *P < 0.05 compared with miR‐NC group. (**D, E**) JAG1 mRNA and protein expression level were examined *via* qRT‐PCR or Western blot following miR‐199b‐5p transfection in ligamentum flavum cells. (**F**) Notch1 and Notch2 protein expression levels were examined *via* Western blot in miR‐199b‐5p transfected ligamentum flavum cells. (**G**) MiR‐199b‐5p binding site in the 3′‐UTRs of TGFB2 and SOX6 are highly conserved among vertebrates. (**H**) A TGFB2 3′‐UTR or a SOX6 3′‐UTR reporter plasmid was co‐transfected in HEK293T cells with either miR‐199b‐5p or mi‐NC and fluorescence was quantified; *P < 0.05 compared with miR‐NC group.

To further examine whether miR‐199b‐5p directly targets JAG1, TargetScan was utilized to forecast potential miR‐199b‐5p targets. Among the candidates, JAG1 was found to have a miR‐199b‐5p binding site in its 3′‐UTR that is greatly conserved among vertebrates (Fig. 4A). Based on this finding, luciferase reporters that had either a WT JAG1 3′‐UTR or a mutant (MUT) JAG1 3′‐UTR, which contained a mutant miR‐199b‐5p binding site, were constructed (Fig. 4B). These results indicated that miR‐199b‐5p binding of the 3′‐UTR of JAG1 repressed luciferase activity when compared to the miR‐NC control group. Additionally, no statistically significant alteration in luciferase activity was observed in the presence of the mutated 3′‐UTR site (Fig. 4C).

To confirm that miR‐199b‐5p regulates osteogenic differentiation in ligamentum flavum cells by targeting JAG1, JAG1 expression levels were detected following miR‐199b‐5p mimic transfection. While JAG1 mRNA expression levels showed no obvious changes (Fig. 4D), protein levels decreased significantly (Fig. 4E), thus suggesting that miR‐199b‐5p regulates JAG1 post‐transcriptionally. Furthermore, miR‐199b‐5p overexpression decreased Notch2 expression (Fig. 4F), similarly to the effect seen following JAG1 knockdown.

Additionally, it was discovered that TGFB2 and SOX6 also contain a miR‐199b‐5p binding site in their 3′‐UTRs (Fig. 4G); thus, these were examined. As shown in Figure 4H, miR‐199b‐5p down‐regulated the 3′‐UTR luciferase activity of both TGFB2 and SOX6, with this effect being slight but still statistically significant.

### JAG1 knockdown could block the effect of miR‐199b‐5p inhibition

**Figure 5:**
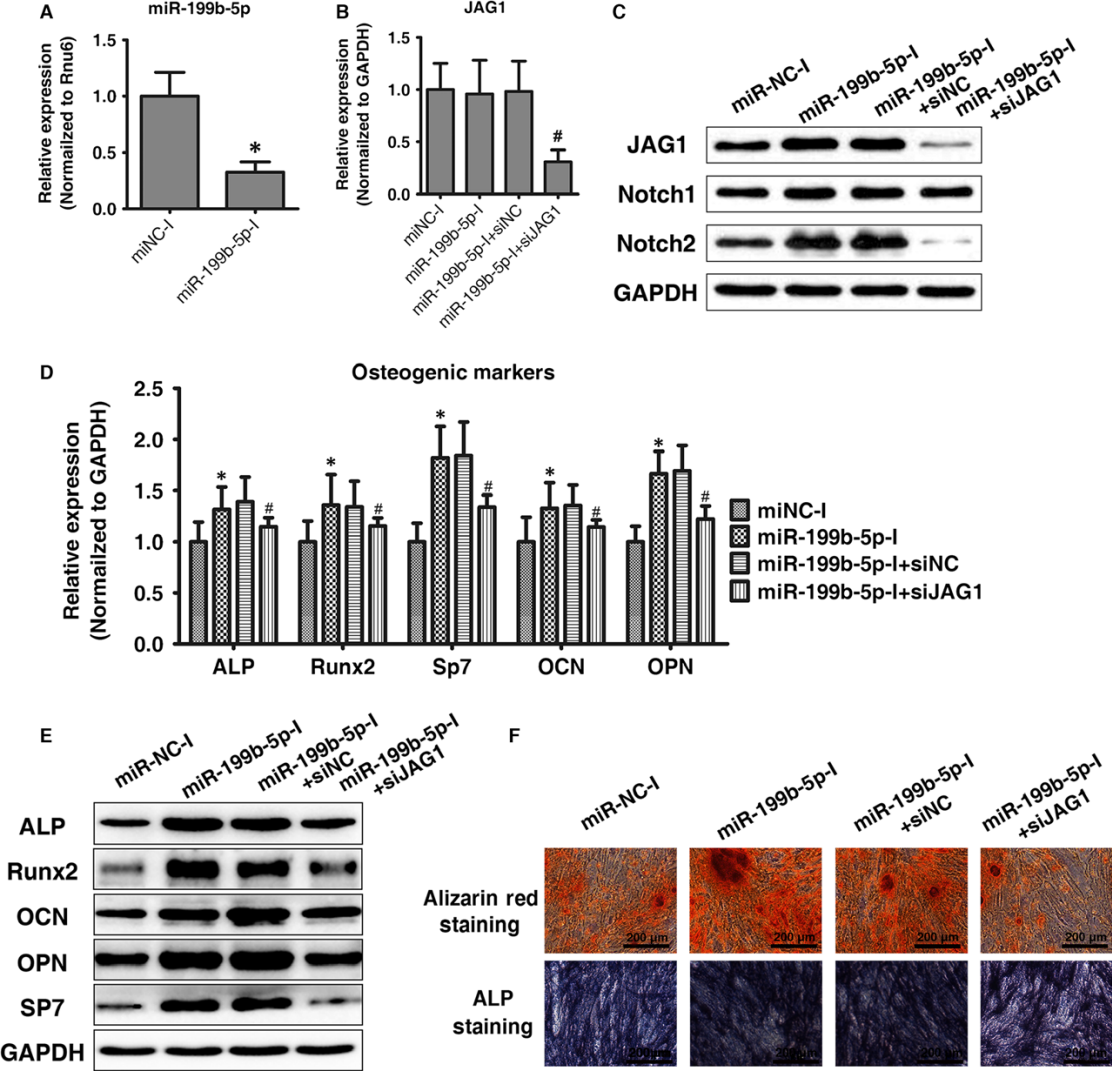
JAG1 knockdown could block the effect of miR‐199b‐5p inhibition. (**A**) MiR‐199b‐5p expression assessed *via* qRT‐PCR in ligamentum flavum cells transfected with miRNA inhibitor; *P < 0.05 compared with miR‐NC‐I group. (**B**) JAG1 mRNA expression levels examined *via* qRT‐PCR following miR‐199b‐5p inhibitor and siJAG1 transfection in ligamentum flavum cells; #*P* < 0.05 compared with ‘miR‐199b‐5p‐I + si‐NC’ group. (**C**) JAG1, Notch1 and Notch2 protein expression levels examined *via* Western blot following miR‐199b‐5p inhibitor and siJAG1 transfection in ligamentum flavum cells. (**D, E**) Osteogenic marker mRNA and protein expression examined *via* qRT‐PCR and Western blot at day 14 after miR‐199b‐5p inhibitor and siJAG1 transfection; *P < 0.05 Compared with ‘miR‐NC‐I’ group. #*P* < 0.05 compared with ‘miR‐199b‐5p‐I + siNC’ group. (**F**) ALP staining and Alizarin Red staining at day 14 following miR‐199b‐5p inhibitor and siJAG1 transfection; scale bar represents 200 μm.

To further confirm that the effect of miR‐199b‐5p during osteogenic differentiation in ligamentum flavum cells is controlled by targeting JAG1, we analysed the effect of miR‐199b‐5p inhibitor on the osteogenic differentiation in ligamentum flavum cells with and without siJAG1. The inhibition rates of miR‐199b‐5p following miR‐199b‐5p inhibitor transfection were shown in Figure 5A. Further as shown in Figure 5B–F, miR‐199b‐5p inhibitor up‐regulated the protein expression of JAG1 and Notch2, and promoted osteogenic differentiation compared to miR‐NC inhibitor. As expected, the effects of miR‐199b‐5p inhibitor on ligamentum flavum cells were abolished after siJAG1 transfected. These results demonstrate that JAG1 knockdown could block the effect of miR‐199b‐5p inhibition, further indicating that miR‐199b‐5p regulated osteogenic differentiation in ligamentum flavum cells through JAG1.

### GSK‐3β/β‐catenin regulates JAG1 and was slightly affected by miR‐199b‐5p in ligamentum flavum cells

**Figure 6:**
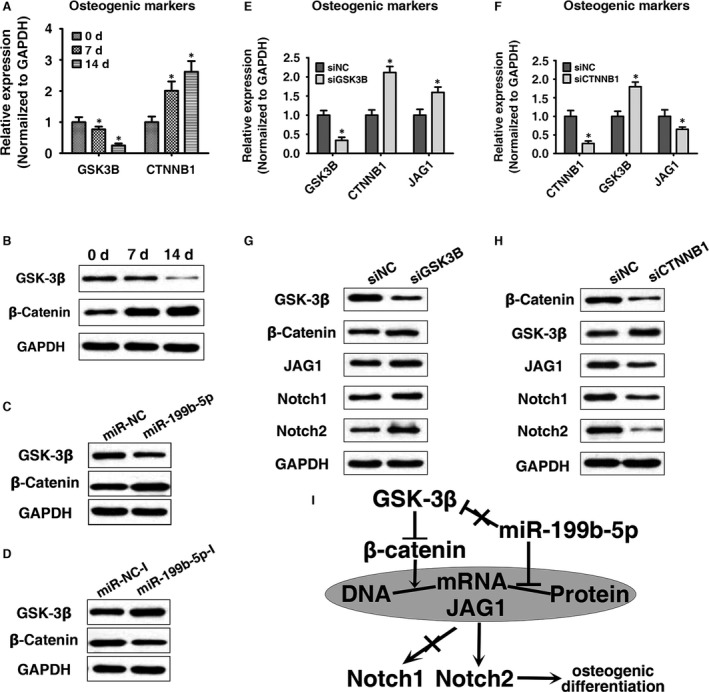
GSK‐3β/β‐catenin regulates JAG1 and was slightly affected by miR‐199b‐5p in ligamentum flavum cells. (**A, B**) GSK‐3β and β‐catenin mRNA and protein expression levels examined *via* qRT‐PCR and Western blot at different time points during osteogenic differentiation in ligamentum flavum cells; *P < 0.05 compared with day 0. (**C, D**) GSK‐3β and β‐catenin protein expression level examined *via* Western blot following miR‐199b‐5p mimic and inhibitor transfection in ligamentum flavum cells. (**E, F**) GSK‐3β, β‐catenin and JAG1 mRNA expression levels examined *via* qRT‐PCR following GSK‐3β and β‐catenin knockdown in ligamentum flavum cells. *P < 0.05 Compared with si‐NC group. (**G, H**) GSK‐3β, β‐catenin, JAG1 Notch1 and Notch2 protein expression levels examined *via* Western blot following GSK‐3β and β‐catenin knockdown in ligamentum flavum cells. (**I**) The regulatory mechanism of osteogenic differentiation in ligamentum flavum cells in this study. ‘⊥’: inhibit; ‘↓’: promote; ‘×’: weak effect.

JAG1 was suggested as a target of β‐catenin 25, and recently it has been shown that miR‐199b‐5p targets the β‐catenin inhibitor GSK‐3β in the BMSCs 22. We next evaluated the potential regulatory mechanism of miR‐199b‐5p/β‐catenin/JAG1 in the ligamentum flavum cells. We first determined the mRNA and protein expression of GSK‐3β and β‐catenin during osteogenic differentiation in ligamentum flavum cells. Results shown in Figure 6A and B suggested that GSK‐3β was down‐regulated while β‐catenin was up‐regulated during osteogenic differentiation. Next, GSK‐3β and β‐catenin expression levels were detected following miR‐199b‐5p mimic or inhibitor transfection. As shown in Figure 6C and D, GSK‐3β protein expression was negatively regulated by miR‐199b‐5p in the ligamentum flavum cells, but the effect was slight. To further elucidate whether β‐catenin targets JAG1 in ligamentum flavum cells, we determined the expression of JAG1 and Notch signalling after GSK‐3β or β‐catenin knockdown. Our results showed that GSK‐3β knockdown increased the β‐catenin expression and up‐regulated JAG1 and Notch signalling (Fig. 6E and G), whereas β‐catenin knockdown led to an opposite effect (Fig. 6F and H). As the mRNA expression of JAG1 was changed following GSK‐3β and β‐catenin knockdown, the regulation of JAG1 expression by β‐catenin might be at the transcription level.

## Discussion

During OLF pathogenesis, fibroblasts differentiate into osteoblasts. When examining patient OLF cells, cells exhibited a fibroblast‐like morphology and expressed fibroblastic markers. Additionally, when these cells were induced to differentiate towards an osteogenic lineage, osteoblastic characteristics were exhibited. Once this process was confirmed, the signalling regulation directing the osteogenic differentiation was examined, with miR‐199b‐5p being of particular interest.

MiR‐199b, which is located on chromosome 9q34.11, has been widely researched in recent years and implicated in tumour progression in tumours such as myeloid leukaemia 26, lymphoid leukaemia 27, medulloblastoma 28, choriocarcinoma 29, endometrioid endometrial carcinoma 30, ovarian cancer 31, breast cancer 32, prostate cancer 33, hepatocellular carcinoma 34, colorectal cancer 35, Kaposi's sarcoma 36, osteosarcoma 37, 38 and Ewing's sarcoma 39. Additionally, miR‐199b has also been implicated in other pathologies such as heart failure 40, idiopathic pulmonary arterial hypertension 41 and intractable epilepsy 42. Furthermore, in a recent integrated microRNA–mRNA study, miR‐199b‐5p was found to be one of the top 10 down‐regulated miRNAs in OPLL cells compared to the normal posterior longitudinal ligament cells 21, thus suggesting that miR‐199b‐5p may be involved in the process of ligament ossification. In the present study, the role of miR‐199b‐5p during osteogenic differentiation in ligamentum flavum cells was examined. These results showed that miR‐199b‐5p was down‐regulated during osteogenic differentiation, while miR‐199b‐5p overexpression inhibited osteogenic differentiation. These findings suggest that miR‐199b‐5p acts as a negative regulator of osteogenic differentiation in ligamentum flavum cells.

Another pathway of interest was the Notch signalling pathway, which has been increasingly implicated as a significant signalling pathway in osteogenesis and bone homoeostasis 43. Notch signalling regulates both chondrogenic and osteogenic differentiation, with aberrant Notch signalling associated with several skeletal disorders, such as osteosclerosis 44, osteoporosis 45, chondrodysplasia 46, osteoarthritis 47, osteosarcoma 48 and congenital diseases or syndromes 49. In our previous study, Notch2, JAG1 and HES1 were found to be up‐regulated in OLF cells, and the Notch signalling pathway was found to promote osteogenic differentiation in ligamentum flavum cells 18.

During cellular differentiation and morphogenesis, JAG1, a Notch1 receptor ligand, binds to its receptor causing the release of the Notch intracellular domain (NICD). The NICD then translocates to the nucleus and activates Notch‐responsive genes 50, 51, 52. JAG1 has also been implicated as an important factor in normal human skeletal development and homoeostasis, with Alagille syndrome, a disease characterized by skeletal abnormalities among other symptoms, caused by a JAG1 gene microdeletion 53. Furthermore, a genome‐wide association study found that the polymorphic JAG1 trait rs2273061 is related to high bone mineral density and low osteoporotic fracture risk 54. In the present study, JAG1 was found to encourage osteogenic differentiation in ligamentum flavum cells, with JAG1 up‐regulated during osteogenic differentiation and JAG1 knockdown inhibiting osteogenic differentiation. Moreover, JAG1 knockdown also inhibited Notch signalling, thus suggesting that osteogenic differentiation is executed through the Notch signalling pathway, with JAG1 being one of the principal Notch ligands. Additionally, JAG1 was found to associate with Notch2, but not Notch1, during osteogenic differentiation in human ligamentum flavum cells, which is consistent with our previous study 18.

While previous studies have reported that miR‐199b‐5p targets Notch signalling in various cell types 28, 31, 37, 38, 55, 56, this study identified JAG1 as a target of miR‐199b‐5p during osteogenic differentiation in ligamentum flavum cells. Moreover, miR‐199b‐5p down‐regulated JAG1 protein expression, thus suggesting post‐transcriptional regulation, and miR‐199b‐5p overexpression decreased Notch signalling. Furthermore, JAG1 knockdown could block the effect of miR‐199b‐5p inhibition. It is well known that one miroRNA can regulate multiple target genes; thus, other potential miR‐199b‐5p targets were predicted using Targetscan. This revealed that other osteogenesis‐related genes, such as SOX6, a crucial transcription factor that regulates endochondral ossification 57, and TGFB2, a member of the transforming growth factor beta family of cytokines 58, were also potential miR‐199b‐5p targets. These proteins were found to exhibit a weak effect following miR‐199b‐5p binding in their 3′‐UTRs; therefore, further research is obligatory to better comprehend how miR‐199b‐5p modulates osteogenic differentiation in ligamentum flavum cells.

β‐catenin signalling pathway was also an important signalling pathway in osteogenic differentiation in ligamentum flavum cells 6, and JAG1 was known as a target of β‐catenin 25, 52, 59. Recently, a study found that miR‐199b‐5p was up‐regulated and targets the β‐catenin inhibitor GSK‐3β in the osteogenic differentiation in the BMSCs 22, which contradict the results presented in this study. We believe the main reason leads to the differences in the two studies is the cell lines that used in two studies. The mechanism of osteogenic differentiation in BMSCs and ligamentum flavum cells may not be completely consistent. Generally, in ligamentum flavum cells, as summarized in Figure 6I, miR‐199b‐5p inhibits JAG1 at post‐transcriptional level and β‐catenin promotes JAG1 at transcription level. Moreover, miR‐199b‐5p has only a slight impact on GSK‐3β/β‐catenin.

Overall, the findings presented herein show that miR‐199b‐5p can suppress osteogenic differentiation in ligamentum flavum cells by targeting JAG1 and inhibiting Notch signalling. Moreover, these findings suggest that JAG1 and miR‐199b‐5p could possibly be viable therapeutic targets for ossification management in ligamentum flavum and other skeletal disorders.

## Funding

This work was supported by the National Natural Science Foundation of China (Grant numbers 81272031 and 81071505).

## Conflict of interest statement

The authors declare no conflicts of interest.
